# Precision Heart Rate Estimation Using a PPG Sensor Patch Equipped with New Algorithms of Pre-Quality Checking and Hankel Decomposition

**DOI:** 10.3390/s23136180

**Published:** 2023-07-05

**Authors:** Smriti Thakur, Paul C.-P. Chao, Cheng-Han Tsai

**Affiliations:** Department of Electronics and Electrical Engineering, National Yang Ming Chiao Tung University, Hsinchu 300, Taiwan

**Keywords:** heart rate (HR), photoplethysmogram (PPG), motion artifact, notch filter, Hankel matrix, singular value decomposition (SVD), beats per minutes (bpm)

## Abstract

A new method for accurately estimating heart rates based on a single photoplethysmography (PPG) signal and accelerations is proposed in this study, considering motion artifacts due to subjects’ hand motions and walking. The method comprises two sub-algorithms: pre-quality checking and motion artifact removal (MAR) via Hankel decomposition. PPGs and accelerations were collected using a wearable device equipped with a PPG sensor patch and a 3-axis accelerometer. The motion artifacts caused by hand movements and walking were effectively mitigated by the two aforementioned sub-algorithms. The first sub-algorithm utilized a new quality-assessment criterion to identify highly noise-contaminated PPG signals and exclude them from subsequent processing. The second sub-algorithm employed the Hankel matrix and singular value decomposition (SVD) to effectively identify, decompose, and remove motion artifacts. Experimental data collected during hand-moving and walking were considered for evaluation. The performance of the proposed algorithms was assessed using the datasets from the IEEE Signal Processing Cup 2015. The obtained results demonstrated an average error of merely 0.7345 ± 8.1129 beats per minute (bpm) and a mean absolute error of 1.86 bpm for walking, making it the second most accurate method to date that employs a single PPG and a 3-axis accelerometer. The proposed method also achieved the best accuracy of 3.78 bpm in mean absolute errors among all previously reported studies for hand-moving scenarios.

## 1. Introduction

The first on-market PPG (photoplethysmogram) sensor was the pulse oximeter, which was commercialized around 1980 for hospital use [1]. This PPG sensor device consists of two alternating LEDs in red/infrared and a photodetector (PD) to obtain information about blood vessel pulsations from the output signals of the PD. The devices are now available on the market in the form of wearable smart watches, earphones, etc. [2]. The non-invasiveness, ease of use, and variety in price, design, and uses have made this industry of wearable smart watches flourish within a few years. However, further advances of this PPG technology are nowadays seriously hampered by the unavoidable contamination of PPG signals by noises mainly from motion artifacts (MAs) [2,3,4,5], which are caused by significantly unavoidable changes in the optical power paths from LEDs and PDs during motions, resulting in undesired components to be measured in the PPG signals. These noises become worse when the sensor is dislocated or in loose contact with the body. With MAs, obtaining the correct heart rate (HR), blood oxygen saturation (SpO_2_), and blood pressure (BP) based on PPGs becomes very difficult and sometimes impossible due to the absence of uncontaminated PPG signals measured from wearable devices.

Many reported studies have been dedicated to detecting, evaluating, mitigating, and/or removing motion artifacts (MAs) from PPG signals. The detection of MAs can be carried out by statistical means. Rajet Krishnan et al. [3] used kurtosis and skewness in both time and frequency domains to distinguish between clean and MA-corrupted data. Some researchers, such as Hanyu et al. [6], have also used the standard deviation and mean error along with selected parameters to detect MAs and remove them. Another method is to consider the spectral analysis of measured raw PPG signals based on ensemble empirical mode decomposition (EEMD) with spectrum subtraction (SS) [7,8]. In this method, corrupted PPG signals are classified into corrupted, moderately corrupted, and clean by the thresholds on the amplitudes and frequencies of dominant peaks. Bashar et al. [9] developed a method of variable frequency complex demodulation (VFCDM) and applied this to a set of 200 subjects for PPGs measured at fingers and wrists as well as with elbow movements. They acquired 449 recordings, with the result that 156 were misclassified and 29 were false-positives. Once detected, the MAs can be removed. Kong et al. [10] also used VFCDM. Some other studies, such as [11,12], conducted motion artifact removal (MAR) with assistance from reference signals from multiple LEDs (blind separation methods, Kalman filtering, etc.) and/or an accelerometer (adaptive filter). With multiple LEDs, Raghuram et al. [11] and Hara et al. [12] showed an accuracy of 0.392 bpm using PPG with finger motions, achieving a RMSE of 6.5 for walking, running, and jumping. On the other hand, using accelerometers, Lin and Ma [13] adopted discrete wavelet transforms for noise reduction. They calculated and tracked heart rates using the Kalman Filter. Mahdi Boloursaz et al. [14] utilized accelerometer signals before applying an LMS filter for MAR. Other techniques include an independent component analysis (ICA), adopted by [15,16,17,18,19]; empirical mode decomposition (EMD), used by [11,20,21,22,23,24,25]; least mean squares (LMS), used by [26,27,28,29]; wavelet transform (WT), used by [30,31,32]; a notch filter, used by [33]; or recently, in 2022, adaptive filters, used by [34]. With advances in technology, some very recent attempts at using machine learning for MAR have also been reported [35,36,37,38,39]. Most recently, in 2023, attention has been turned to lightweight machine learning modules [40] that are implementable in wearable devices.

Different from all the previous studies, this study proposes two sub-algorithms in a series to remove motion artifacts, which were applied to cases of hand movements and walking. The first was a new quality-assessment criterion to disregard highly noise-contaminated PPG signals, while the second employed the Hankel matrix and the associated SVD to remove motion artifacts. The aforementioned pre-screening of noises by the first quality-check sub-algorithm, and the capability of the subsequent Hankel matrices and the associated SVD to identify, decompose, and remove the motion components in measured PPG were expected to deliver a high-precision HR estimation. Two cases of walking and hand movement were considered to demonstrate the effectiveness of the proposed MAR algorithm. The IEEE Signal Processing Cup 2015 dataset was used for performance evaluation. The average error result was 0.7345 ± 8.1129 bpm, with a mean absolute error (MAE) of 1.86 bpm (beats per minute); the second-best of all the reported results. As for hand-moving, this study showed the best accuracy of 3.78 bpm in MAE compared with all the reported studies.

The remainder of this article is organized into four sections. Section 2 describes how the PPG signals were collected. Section 3 details the first algorithm of the quality check on measured PPG signals, while Section 4 describes the algorithm of motion artifact removal using the Hankel matrix and the associated SVD. Section 5 presents the experimental validation of the performance of the proposed method. Finally, Section 6 concludes this study.

## 2. PPG Signals and Motion Artifacts

### 2.1. Measuring PPG

**Figure 1:**
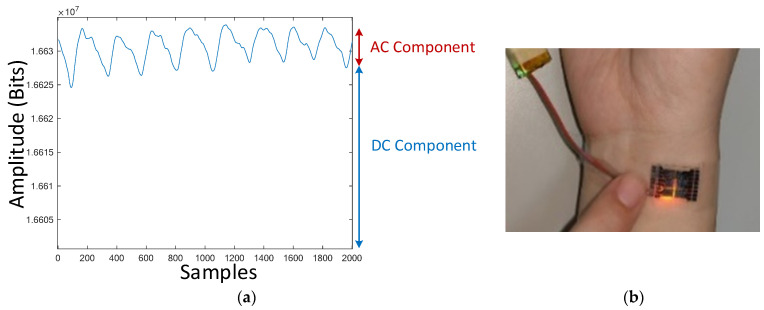
(**a**) AC and DC components of a typical PPG signal; (**b**) PPG sensor patch developed by [41].

**Figure 2:**
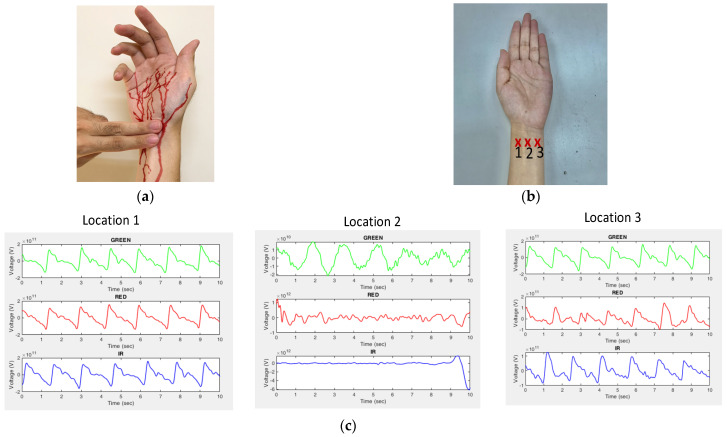
(**a**) Human arteries at wrist; (**b**) three different locations on the wrist to measure PPG; (**c**) measured PPGs using green, red, and infrared LEDs at three different locations.

**Figure 3:**
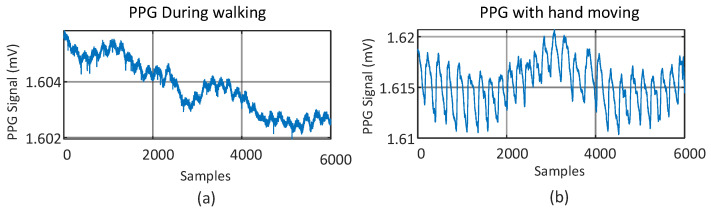
PPGs measured at a wrist artery contaminated by two different motion artifacts: (**a**) walking; (**b**) hand movement.

A typical PPG signal at the wrist artery, where most wearable devices measure PPG, is shown in Figure 1a, which was measured by the PPG sensor patch developed exclusively by [41], as given in Figure 1b. This typical PPG waveform consists of AC and DC components. The AC component reflects the pulsatile component of blood vessel pulsations synchronized with heart beats, while the DC component is the non-pulsatile component resulting from light absorption in tissue, skin, and bones along the optical paths between LEDs and PD of the PPG devices. To find the best wavelength of LEDs and the best location to measure PPG, a series of experiments with the subject sitting still were conducted with the PPG sensor in Figure 1 emitted at different wavelengths and attached at different locations 1–3, as illustrated in Figure 2a–c. It can be seen from Figure 2a–c that both green and infrared (IR) LEDs lead to larger AC components, while locations 1 and 3 lead to larger ACs in the measured PPGs as opposed to others. This is, in fact, due to the presence of arteries under the locations as shown in Figure 2a, like seen in [42]. For the remainder of this study, to develop the algorithm for removing motion artifacts, the PPG sensor patch in Figure 1b is attached at location 1, as seen in Figure 2b, to obtain the PPG measured from the green LED. Typical PPGs contaminated by motion artifacts, measured by the green LED at location 1 on the wrist artery, are shown in Figure 3a,b, which are those during walking and with hand motion, respectively. Based on the comparison between Figure 3a,b, it can be seen that the PPG waveforms measured with hand movement exhibit much smaller DC drifts than walking, while the AC components of both cases are close to each other. Additionally, the large DC drifts, especially in the case of walking, present a serious, negative effect of motion artifacts on estimating the heart rate, blood oxygen, and/or blood pressure based on measured PPG waveforms. 

### 2.2. Classification of Motion Artifacts 

**Figure 4:**
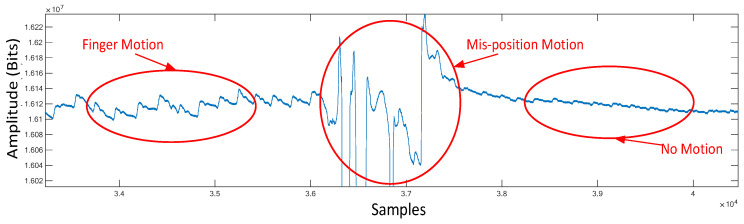
PPG contaminated by motion and mispositioning.

**Figure 5:**
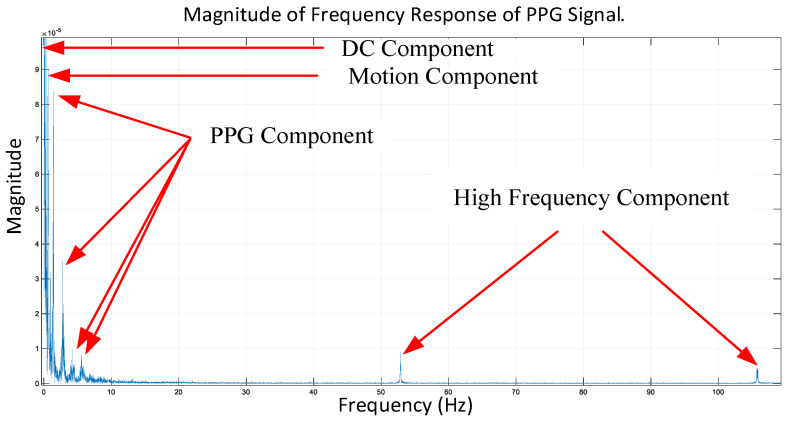
Frequency response of the measured PPG signal.

Motion artifacts are understood nowadays as having two different magnitudes, micro and macro motions, as shown in Figure 4. The micro-motion artifacts have been defined as those due to measuring position adjusting, gesture changing, or finger tapping, while the macro-motion artifacts are those having consistent body motion, such as walking, jogging, and running. Figure 4 shows the measured PPGs contaminated by the micro- and macro-motion artifacts due to finger tapping, measuring position adjusting (both leading to micro-MAs), and walking (macro-MAs). It can be seen from this figure that both micro- and macro-motion artifacts cause much more significant fluctuations to the measured PPG signal than sedentary gestures of the subject. All the aforementioned motions could range between 0.1 and 20 Hz, which is right within the frequency range for heart rate (1–4 Hz), causing much trouble for estimating heart rate based on measured PPG signals. Figure 5 shows the frequency response of a contaminated PPG signal with a few components away from the given frequencies. With the presence of micro and macro motions, the estimation accuracy of heart rate could be seriously compromised. To remedy this problem, two algorithms are proposed herein for motion artifact reduction (MAR), as described in the ensuing Section 3 and Section 4.

## 3. The First Sub-Algorithm of Quality Check on Measured PPG Signals

**Figure 6:**
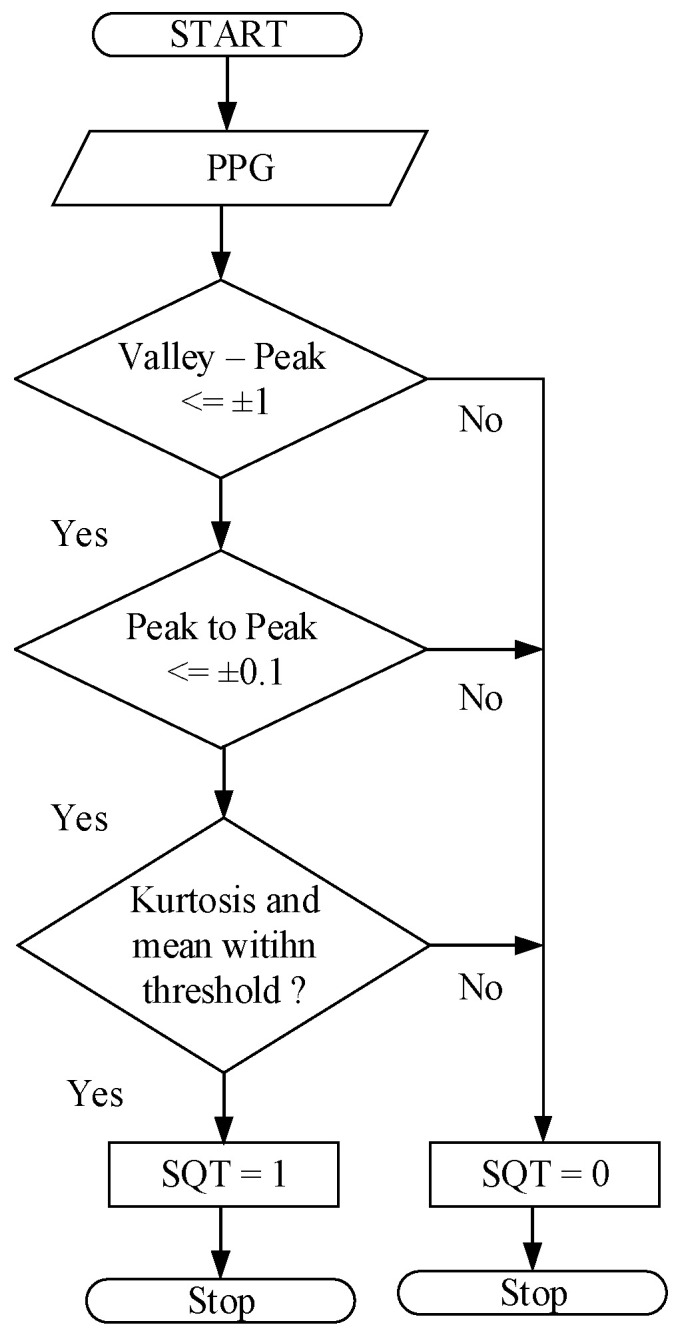
Computation flow of the quality-check algorithm on the measured PPG.

The algorithm proposed by this study consists of two sub-algorithms to increase the accuracy of heart rate estimation with serious motion artifacts in PPGs measured by the wearable device. The first sub-algorithm is designed to check the quality of real-time raw PPGs measured to rule out those seriously contaminated and keep the information of heart rate still embedded in the measured PPGs. With this first sub-algorithm, the accuracy of estimating heart rate is expected to increase. To this end, a new algorithm for signal quality checking on measured PPG has been developed. Figure 6 elaborates on its computation flow. Note that a qualified PPG signal at the end is defined as the one that is periodic in time domain and has the largest frequency component remaining within a limited range close to that of the legitimate heart rate. Accordingly, specific conditions in a sequence for checking are defined below for subsequent analysis to extract precision heart rate in the next section.

**Figure 7:**
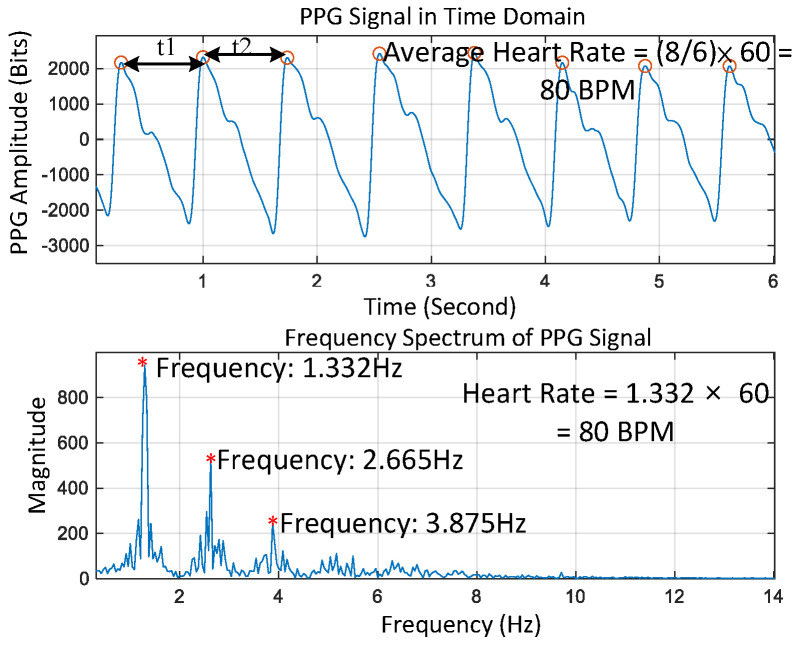
Measured PPG signal in time and frequency domains.

**Figure 8:**
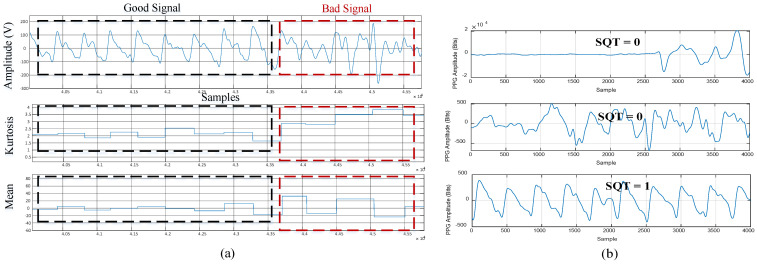
(**a**) Mean and kurtosis for qualified and unqualified PPG signals; (**b**) SQT for the PPG signal.

(1) The number of valleys and peaks of the measured PPG signal is calculated, and then it is checked if
  (1) (Number of Valleys) ≤ (Number of Peaks)±1,
  to affirm the presence of pulsation in the measured PPG; otherwise, the signal is labeled as “unqualified” and we then return to stage one for motion artifact reduction.
(2) The *t*_i_ values are defined as the time intervals between peaks that can be extracted from measured PPGs, as shown in Figure 7. It is next checked if all differences between consecutive *t*_i_ values,
  Δ*t* = *t*_i+1_ − *t*_i_, (2)
  are each less than or equal to ±0.1 of *t*_i_. If this condition is true, the signal is labelled as “qualified,” and then we go to the next step in Figure 6; otherwise, the signal is labelled as “unqualified” and we stop the computation.
(3) The statistical measures of kurtosis, mean, and standard deviation of the qualified PPG segments are calculated further at each cycle. If all calculated statistical values are within pre-defined thresholds, as shown in Figure 8a, the measured PPG is identified as “qualified;” otherwise, it is determined as as “unqualified” and then the computation is stopped. Note that a similar approach was used by [3] for motion artifact detection.
(4) A marker SQT (Signal Quality Token) is defined as either ’0’ or ‘1’ to label the measured PPG segment as qualified or unqualified. Figure 8b shows three representative examples of PPG marked with different SQTs. Only the PPG with SQRT = 1 is passed on to calculate heart rate based on the Hankel matrix and SVD decomposition.

## 4. The Second Sub-Algorithm for Motion Artifact Removal

The PPG signals determined as “qualified” by the first sub-algorithm of quality checking in the previous section are next processed with the motion artifact removal (MAR) algorithm proposed herein.

### 4.1. The Hankel Matrix and Its SVD

**Figure 9:**
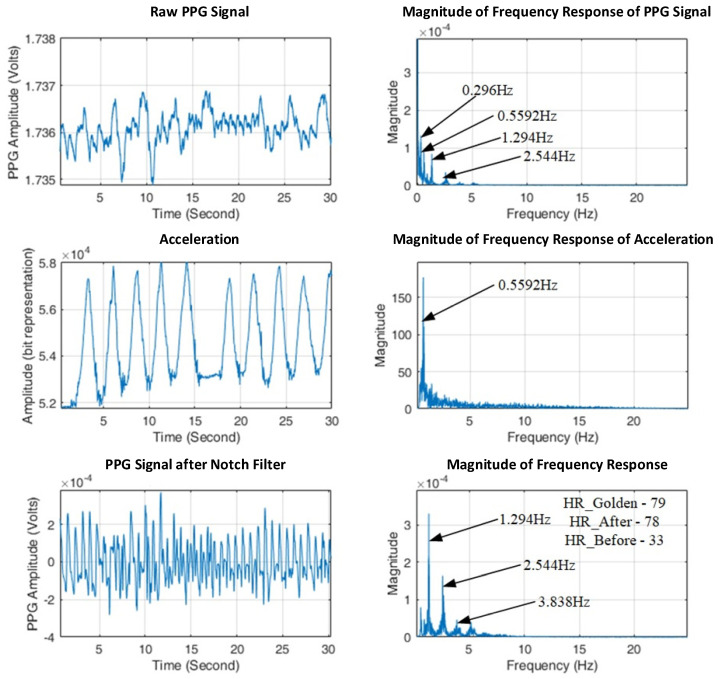
Estimating heart rate for hand movement using the proposed algorithms.

Precision estimation of heart rate is carried out herein by singular value decomposition on the Hankel matrix of the measured PPG and motion via an accelerometer also attached to the PPG sensor, as shown in Figure 1b. The PPG and accelerometer signals are measured and recorded for six seconds and then first organized into Hankel matrices [43], **H**_ppg_, **H**_x_, **H**_y_, and **H**_z_, respectively, for PPG and accelerations along x, y, and z. For example, **H**_ppg_ is in the form of

(3) $$H_{\mathrm{ppg}}=\left[\begin{array}{cccc} a(t_{1}) & a(t_{2}) & \ldots & a(t_{n})\\ a(t_{2}) & \ddots & & \vdots \\ \vdots & & \ddots & \vdots \\ a(t_{n}) & \cdots & \cdots & a(t_{2n}) \end{array}\right],$$

where *a*(*t*)’s are PPG data at times of *t*’s, and there are in total 2*n* PPG data in the sampled window of six seconds. Next, the single value decompositions (SVDs) [44] are conducted on each of **H**_ppg_, **H**_x_, **H**_y_, and **H**_z_, leading to

(4) $$H_{\mathrm{ppg}}=U_{\mathrm{ppg}}\Sigma_{\mathrm{ppg}}V^{T}{}_{\mathrm{ppg}},$$

(5) $$H_{x}=U_{x}\Sigma_{x}V_{x}{}^{T},$$

(6) $$H_{y}=U_{y}\Sigma_{y}V_{y}{}^{T},$$

(7) $$\mathrm{and}\;H_{z}=U_{z}\Sigma_{z}V_{z}{}^{T}$$

respectively, where **U**, **Σ**, and **V** correspond to orthogonal, diagonal, orthogonal matrices. **U** contains the orthogonal basis for the column space of **H**, while **V** contains the orthogonal basis for the row space of **H**. **Σ** contains the eigenvalues of matrix **H**. The eigenvalues of the diagonal matrices **Σ** are stored as **Λ**_ppg_, **Λ**_x_, **Λ**_y_, and **Λ**_z_ for further processing. In the next steps, the eigenvalues with the associated components highly correlated to motion artifacts are removed. To this end, the correlation matrix of PPG and 3-axis accelerometer signals can be calculated; that is [45],

(8) $$\rho_{x,y}=\mathrm{corr}(x,y)=\frac{\mathrm{cov}(x,y)}{\sigma_{x}\sigma_{y}}=\frac{E(x-\mu_{x})\;E(y-\mu_{y})}{\sigma_{x}\sigma_{y}},$$

where the correlation value *ρ***_x,y_** gives the similarity index between the two signals, with expected values *μ***_x_** and *μ***_y_**, and standard *σ***_x_** and *σ***_y_** of the two signals. The components in measured PPG that are highly correlated to accelerations are removed. Then, the time-domain PPG signal without motion artifacts can be restored by recovering the frequency response with the non-MR-related components only via the inverse Fourier transform, as seen in Figure 9.

### 4.2. The Computational Flow of the Proposed MAR Algorithm

**Figure 10:**
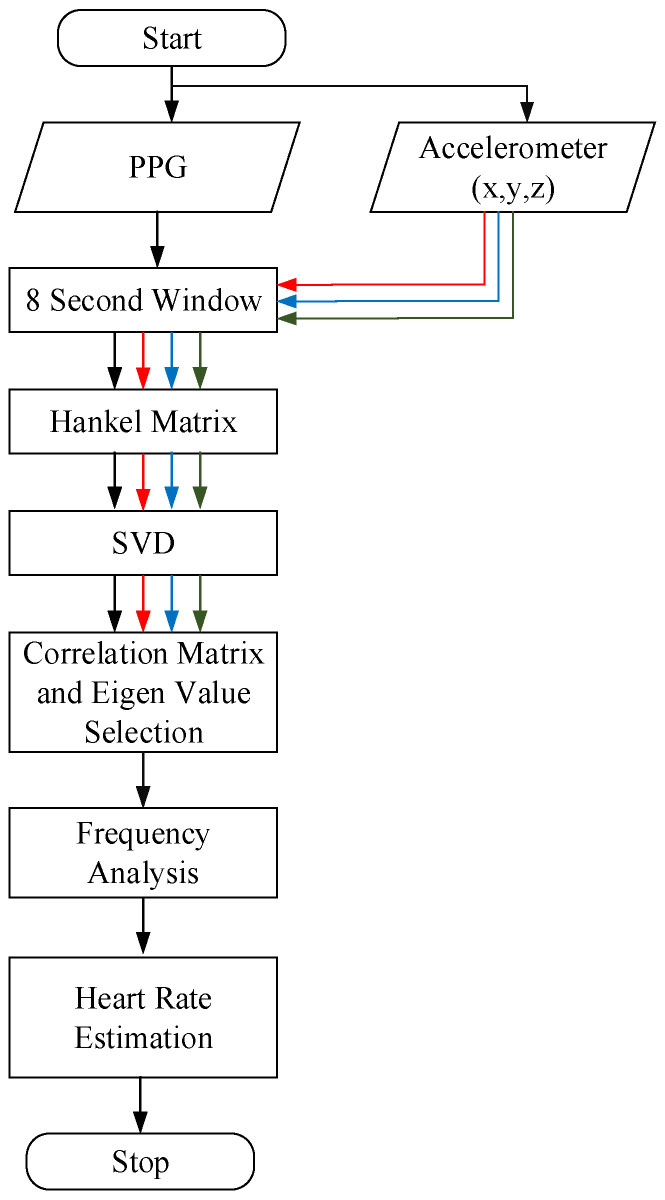
Computation flow of the proposed second sub-algorithm.

A new algorithm of motion artifact removal (MAR) is engineered herein for re-constructing MR-free PPG signals based on the decomposed Hankel matrices in Equations (5)–(7) and their mutual correlation in Equation (8). The associated computation flow is shown in Figure 10, while the pseudo-code is given below. The algorithm (Algorithm 1 in pseudocode) consists of five stages, (a) synthesizing the Hankel matrices **H**_ppg_, **H**_x_, **H**_y_, and **H**_z_ in the forms of (3); (b) Conducting singular value decomposition (SVD) on **H**’s; (c) Calculating correlation to remove MR-related components; (d) Conducting discrete Fourier transform on the MR-free PPG signals and then finding the heart rate by taking the maximum peak of the spectrum as the heartbeat component; (e) Finally, the heart rate is estimated again to see if the consecutive estimated heart rates are close to each other to ensure the robustness of the algorithm.
**Algorithm 1 In pseudocode: Motion Artifact Algorithm for Walking.**1: **Procedure** Record PPG signal and accelerometer signal x,y,z for 8 s 2: Initialize HR_est = 78 3: Construct Hankel matrix Hppg for PPG 4: Construct Hankel matrix Hx for x 5: Construct Hankel matrix Hy for y 6: Construct Hankel matrix Hz for z 7: Find SVD of matrix obtained from step 3, 4, 5, 6 8: Construct a correlation matrix between the 3-axis accelerometer and PPG 9: Select eigenvalues 10: Reconstruct using inverse SVD 11: Find DFT of the reconstructed signal 12: Find HR 13: Heart rate estimation 14: **End procedure**

## 5. Experimental Results

**Figure 11:**
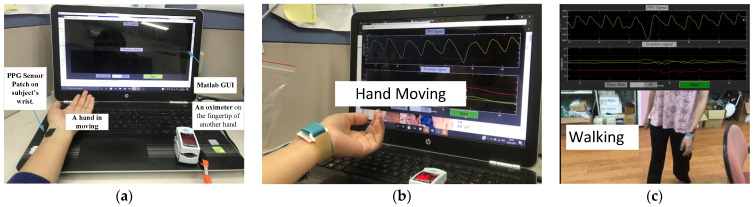
(**a**) Experiment setup for (**b**) hand movement and (**c**) walking.

Experiments were conducted for the cases of hand movement and walking, as shown in Figure 11, to validate the performance of the two sub-algorithms built. A commercially certified oximeter was utilized to provide reference heart rates for validating the performance of the two designed sub-algorithms. All experiments for collecting data were conducted at room temperature, 28~31 °C. The skin temperature of the subject during the experiment is noted to be 35~37 °C. The PPG signals and the accelerometer signals are recorded simultaneously using a printed circuit board (PCB). A Bluetooth connection was established, allowing the subject to freely move his/her arm and walk.

### 5.1. Hand Movement

**Figure 12:**
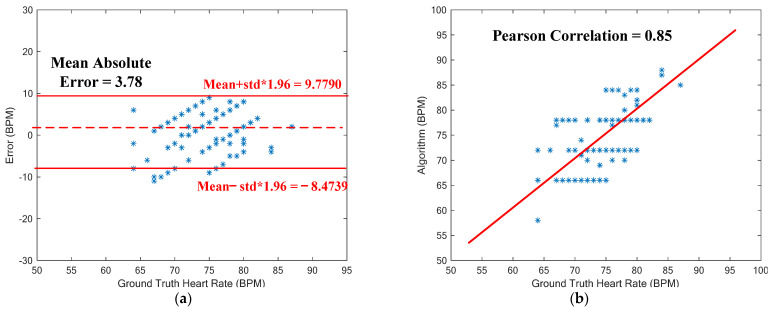
(**a**) Bland–Atman plot for heart rate estimation. (**b**) Correlation plot of estimated HRs vs. ground-truth HRs for the case of hand movement.

The experiment setup with hand movement is shown in Figure 11a,b. A PPG sensor was attached to the wrist artery of a subject, while the fingertip of another hand was clamped with the aforementioned oximeter for reference. The subjects were asked to sit and relax for some time, and then their information, such as skin color, temperature, and reference heart rate from the oximeter, was recorded and noted. Then the subjects were allowed to move the hand up and down with the PPG sensor patch worn at the wrist. The algorithm (consisting of two sub-algorithms) was tested on 10 subjects of three different skin tones (beige, honey, and bronze). The results are shown in Figure 12. The proposed system achieves an accuracy of 0.6525 ± 4.7 bpm with a window of 6 s. The Pearson correlation is 0.6, while the average absolute error (MAE) is 3.78 beats per minute (bpm).

### 5.2. Walking

**Figure 13:**
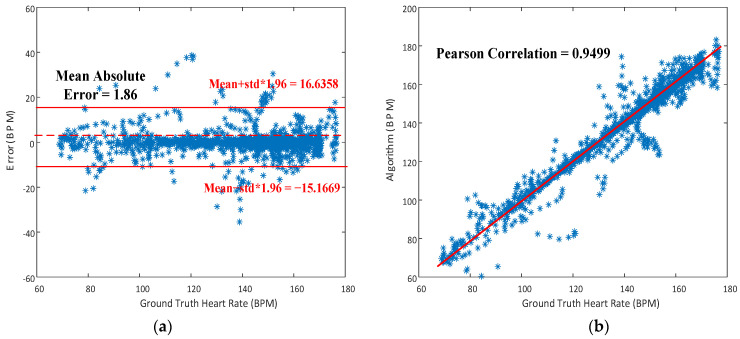
(**a**) Bland-Atman plot for heart rate estimation; (**b**) Correlation plot of estimated HRs vs ground-truth HRs for the case of walking.

The proposed MAR algorithm for walking was tested again on these 10 subjects for walking, as seen in Figure 11c, while the datasets of the IEEE Signal Processing Cup 2015 were used to evaluate the performance of the developed algorithms. The results are shown in Figure 13. Seen in Figure 13a is the Bland–Altman plot of the results predicted by the proposed algorithm for 8-s windows of walking. The resulting average error is as low as 0.7345 ± 8.1129 bpm (beats per minute), with a mean absolute error (MAE) of 1.86 bpm. The associated Pearson correlation is 0.9499, as seen in Figure 13b. Note herein that the MAE of 1.86 bpm for walking being lower than 3.78 bpm for hand-moving is due to much smaller hand-waving amplitudes during walking than intentional hand movement of the subjects.

### 5.3. Discussion

The performance of the proposed two sub-algorithms is compared herein to the results delivered by other reported past studies. Table 1 [6,13,19] and Table 2 [8,10,22,23,24,25,29,46] show the comparison among the algorithms for hand movement and walking, respectively. The mean absolute error (MAE) is considered the main metric for performance comparison. Table 1 gives the performance comparison with other reported studies on MAR for hand movement, while Table 2 does for walking. It can be seen from Table 1 that the present study shows the best accuracy of 3.78 bpm in mean absolute error (MAE) as compared to all the reported studies. The reasons that the present study renders better results compared to other studies [6,13] are the use of the accelerometer signals as a reference and the much better quality checking offered by this work. In comparison to [19], this work identifies the pulsation from PPG before estimating HR, while the work in [19] relies only on a single accelerometer. 

As for walking, the mean absolute error (MAE) offered by our algorithm for walking is 1.86 bpm (beats per minute), the second best but very close to the best [46] of all the reported results. Among all the studies in Table 2, most of them [8,10,22,23,29,46] render better results, with MAEs under 3 bpm, where two-channel PPGs and an accelerometer are employed for HR estimation, the same as in this study, to achieve favorable performance. On top of these studies are those [29,46] that achieve MAEs under 2 bpm for walking. Arunkumar and Bhaskar in 2020 [29] achieved an MAE of 1.89 bpm by developing the recursive least squares (RLS) and normalized least mean squares (NLMS) adaptive filters to remove MAs in the frequency domain. As for the work by Motin et al. in 2019 [46], recursive Wiener filtering was employed. Since Wiener filtering has been considered effective in removing MAs by many studies, the result showed a very favorable low error of HR estimation of 1.85 bpm. Even with very low MAEs [29,46], it could be difficult to remove MAs accurately if the frequency of motion (walking) is close to HR. The methods of Hankel and SVD proposed by this study are based on correlations, which are supposedly more capable of identifying and then removing MAs in the frequencies close to HR. In a nutshell, the favorable precision of HR estimation is due to the pre-screening of noises by the quality-check sub-algorithm and the capability of the subsequent Hankel matrices and the associated SVD to identify, decompose, and remove the components in measured PPGs that are highly correlated to motions.

## 6. Conclusions

Effort was dedicated to developing new algorithms for motion artifact removal (MAR) to accurately estimate heart rate when the subject is in motion, such as hand movement and walking. The proposed algorithm consists of two sub-algorithms. The first is a new quality-assessment criteria to disregard highly noise-contaminated PPG signals, while the second employs the Hankel matrix and SVD to remove motion artifacts. The second sub-algorithm for MAR (motion artifact removal) is built upon (1) assembling Hankel matrices of PPG and accelerations and (2) singular value decomposition to remove the frequency components related to motions for accurate heart rate estimation. The result shows a mean absolute error (MAE) of 1.86 bpm (beats per minute) for walking, the second best but close to the best among all the reported results. As for hand movement, the algorithm shows the best accuracy of 3.78 bpm in MAE as compared to all the other reported results.

## Figures and Tables

**Table 1:** Comparison among various techniques for MAR on hand movement.

	Year	Technique	Sensors (Database)	Reference Signal	Movement	Mean Absolute Error in (bpm)	Mean Error (bpm)	Measurement Location
This study	2023	Quality Check and Notch Filtering with peak selection and current and gain tuning	One-channel PPG and 3-axis accelerometer signals recorded in the lab	Accelerometer	Waving the hand	**3.78** 95% of HR estimation within ± 9.3 bpm	0.6525	Wrist
Lin and Ma [13]	2016	DWT	PPG signals	None	Waving the hand	6.87	NA	NA
Hanyu and Xiao hui [6]	2017	Statistical Evaluation	PPG signals	None	Finger tapping or hand swinging	7.85	NA	NA
Chao Zhao et al. [19]	2021	ICA, VMD, WSST, SSA, and Kalman Smoothing	A three-axis acceleration signals	None	Finger tapping or hand swinging	95% of HR estimation within ±8.86 bpm	NA	Wrist

**Table 2:** Performance comparison among various techniques for MAR on walking.

	Year	Technique	Sensors (Database)	Reference Signal	Movement	Mean Absolute Error (bpm)	Mean Error (bpm)	Measurement Location
This Study	2023	Hankel Matrix, SVD and Spectral Analysis	Two-channel PPG signals, three-axis accelerations	Accelerometer and a single PPG	Walking	**1.86**	0.7345	Wrist
Amirhossein Koneshloo et al. [22]	2019	Joint Basis Pursuit Linear Program	Two-channel PPG signals, three-axis accelerations	Accelerometer and PPG signal.	Walking and running	2.61	NA	Wrist
Mohammod Abdul Motin et al. [46]	2019	Recursive Wiener Filtering	Two-channel PPG signals, three-axis accelerations	Accelerometer and PPG signal	Walking and running	1.85	NA	Wrist
Wenwen He et al. [23]	2020	Motion tracking, Sparse Representation-based MA elimination, and Spectral Peak Tracking for HR	PPG signals with 3-axis accelerometer signal	Accelerometer	Quasi-periodic motions.	2.40	NA	Wrist
Deniz Alp Savaskan et al. [24]	2020	SPECMAR, TROIKA and JOSS methods along with pre and post processing	Two-channel PPG signals, three-axis acceleration signals for 12 samples	Accelerometer and PPG signal	Walking and running	4.19	NA	Wrist
Youngsun Kong et al. [10]	2019	VFCDM approach, Cubic Spline	Two-channel PPG signals, three-axis acceleration signals	Accelerometer and PPG signal	Walking and running	2.94	NA	Wrist
			Two-channel PPG signals, three-axis acceleration signals (lab)	Accelerometer and PPG signal	Walking and running		NA	Forehead
Nicholas Huang et al. [25]	2020	TAPIR Method	Two-channel PPG signals, three-axis acceleration signals	Accelerometer and PPG signal	Walking	9.21	NA	Wrist
K.R. Arunkumar et al. [29]	2020	Recursive Least Squares (RLS) and Normalized Least Mean Squares (NLMS)	Two-channel PPG signals, three-axis acceleration signals recorded for 23 samples	Accelerometer and PPG signal.	Walking and running	1.89	NA	Wrist
S. Friman et al. [8]	2022	Electromyogram (EMG) and accelerometer (ACC)	PPG signals with three-axis acceleration signals	Accelerometer, EMG and PPG signal	Walking and running	2.83	NA	Wrist

## Data Availability

The data presented in this study is available on request from the corresponding author. The data is not publicly available due to privacy and ethical restrictions.

## References

[1] Zenian A. Technology Spotlight: Taking the Pulse of the Pulse Oximetry Market. https://24x7mag.com/medical-equipment/patient-care-equipment/technology-spotlight-taking-pulse-pulse-oximetry-market/.

[2] Castaneda D., Esparza A., Ghamari M., Soltanpur C., Nazeran H. (2018). A review on wearable photoplethysmography sensors and their potential future applications in health care. Int. J. Biosens. Bioelectron..

[3] Krishnan R., Natarajan B., Warren S. Analysis and detection of motion artifact in photoplethysmographic data using higher order statistics. Proceedings of the 2008 IEEE International Conference on Acoustics, Speech and Signal Processing.

[4] Lee Y., Shin H., Jo J., Lee Y.-K. Development of a wristwatch-type PPG array sensor module. Proceedings of the 2011 IEEE International Conference on Consumer Electronics-Berlin (ICCE-Berlin).

[5] Thomas S.S., Nathan V., Zong C., Soundarapandian K., Shi X., Jafari R. (2015). BioWatch: A noninvasive wrist-based blood pressure monitor that incorporates training techniques for posture and subject variability. IEEE J. Biomed. Health Inform..

[6] Shao H.Y., Chen X.H. Motion artifact detection and reduction in PPG signals based on statistics analysis. Proceedings of the 2017 29th Chinese control and decision conference (CCDC).

[7] Zhang Y., Liu B., Zhang Z. (2015). Combining ensemble empirical mode decomposition with spectrum subtraction technique for heart rate monitoring using wrist-type photoplethysmography. Biomed. Signal Process. Control.

[8] Friman S., Vehkaoja A., Perez-Macias J.M. (2022). The use of wrist EMG increases the PPG Heart Rate accuracy in smartwatches. IEEE Sens. J..

[9] Bashar S.K., Han D., Soni A., McManus D.D., Chon K.H. Developing a novel noise artifact detection algorithm for smartphone PPG signals: Preliminary results. Proceedings of the 2018 IEEE EMBS International Conference on Biomedical & Health Informatics (BHI).

[10] Kong Y., Chon K.H. (2019). Heart rate tracking using a wearable photoplethysmographic sensor during treadmill exercise. IEEE Access.

[11] Raghuram M., Sivani K., Reddy K.A. Use of complex EMD generated noise reference for adaptive reduction of motion artifacts from PPG signals. Proceedings of the 2016 International Conference on Electrical, Electronics, and Optimization Techniques (ICEEOT).

[12] Hara S., Shimazaki T., Okuhata H., Nakamura H., Kawabata T., Cai K., Takubo T. Parameter optimization of motion artifact canceling PPG-based heart rate sensor by means of cross validation. Proceedings of the 2017 11th International Symposium on Medical Information and Communication Technology (ISMICT).

[13] Lin W.-J., Ma H.-P. A physiological information extraction method based on wearable PPG sensors with motion artifact removal. Proceedings of the 2016 IEEE International Conference on Communications (ICC).

[14] Mashhadi M.B., Asadi E., Eskandari M., Kiani S., Marvasti F. (2015). Heart rate tracking using wrist-type photoplethysmographic (PPG) signals during physical exercise with simultaneous accelerometry. IEEE Signal Process. Lett..

[15] Lee J., Kim M., Park H.-K., Kim I.Y. (2020). Motion artifact reduction in wearable photoplethysmography based on multi-channel sensors with multiple wavelengths. Sensors.

[16] Kim B.S., Yoo S.K. (2006). Motion artifact reduction in photoplethysmography using independent component analysis. IEEE Trans. Biomed. Eng..

[17] Lee H., Chung H., Ko H., Lee J. (2018). Wearable multichannel photoplethysmography framework for heart rate monitoring during intensive exercise. IEEE Sens. J..

[18] Xiong J., Cai L., Jiang D., Song H., He X. (2016). Spectral matrix decomposition-based motion artifacts removal in multi-channel PPG sensor signals. IEEE Access.

[19] Zhao C., Zeng W., Hu D., Liu H. (2021). Robust heart rate monitoring by a single wrist-worn accelerometer based on signal decomposition. IEEE Sens. J..

[20] Yousef Q., Reaz M., Ali M.A.M. (2012). The analysis of PPG morphology: Investigating the effects of aging on arterial compliance. Meas. Sci. Rev..

[21] Motin M.A., Karmakar C.K., Palaniswami M. (2017). Ensemble empirical mode decomposition with principal component analysis: A novel approach for extracting respiratory rate and heart rate from photoplethysmographic signal. IEEE J. Biomed. Health Inform..

[22] Koneshloo A., Du D. (2019). A novel motion artifact removal method via joint basis pursuit linear Program to accurately monitor heart rate. IEEE Sens. J..

[23] He W., Ye Y., Lu L., Cheng Y., Li Y., Wang Z. (2019). Robust heart rate monitoring for quasi-periodic motions by wrist-type PPG signals. IEEE J. Biomed. Health Inform..

[24] Savaskan D.A., Mahanoglu A., Soner B., Kholmatov A. Heart Rate Measurement from Motion Compensated Photoplethysmographic Signals. Proceedings of the 2020 28th Signal Processing and Communications Applications Conference (SIU).

[25] Huang N., Selvaraj N. Robust ppg-based ambulatory heart rate tracking algorithm. Proceedings of the 2020 42nd Annual International Conference of the IEEE Engineering in Medicine & Biology Society (EMBC).

[26] Temko A. (2017). Accurate heart rate monitoring during physical exercises using PPG. IEEE Trans. Biomed. Eng..

[27] Tanweer K.T., Hasan S.R., Kamboh A.M. Motion artifact reduction from PPG signals during intense exercise using filtered X-LMS. Proceedings of the 2017 IEEE International Symposium on Circuits and Systems (ISCAS).

[28] Wu C.-C., Chen I.-W., Fang W.-C. An implementation of motion artifacts elimination for PPG signal processing based on recursive least squares adaptive filter. Proceedings of the 2017 IEEE Biomedical Circuits and Systems Conference (BioCAS).

[29] Arunkumar K., Bhaskar M. (2020). Robust de-noising technique for accurate heart rate estimation using wrist-type PPG signals. IEEE Sens. J..

[30] Zhang Y., Song S., Vullings R., Biswas D., Simões-Capela N., Van Helleputte N., Van Hoof C., Groenendaal W. (2019). Motion artifact reduction for wrist-worn photoplethysmograph sensors based on different wavelengths. Sensons.

[31] Bousefsaf F., Maaoui C., Pruski A. (2013). Continuous wavelet filtering on webcam photoplethysmographic signals to remotely assess the instantaneous heart rate. Biomed. Signal Process. Control.

[32] Teng X., Zhang Y. Continuous and noninvasive estimation of arterial blood pressure using a photoplethysmographic approach. Proceedings of the 25th Annual International Conference of the IEEE Engineering in Medicine and Biology Society (IEEE Cat. No. 03CH37439).

[33] Zhang Q., Xie Q., Wang M., Wang G. Motion artifact removal for PPG signals based on accurate fundamental frequency estimation and notch filtering. Proceedings of the 2018 40th Annual International Conference of the IEEE Engineering in Medicine and Biology Society (EMBC).

[34] Guo J., Chen X., Zhao J., Zhang X., Chen X. (2022). An Effective Photoplethysmography Heart Rate Estimation Framework Integrating Two-Level Denoising Method and Heart Rate Tracking Algorithm Guided by Finite State Machine. IEEE J. Biomed. Health Inform..

[35] Shuzan M.N.I., Chowdhury M.H., Hossain M.S., Chowdhury M.E., Reaz M.B.I., Uddin M.M., Khandakar A., Mahbub Z.B., Ali S.H.M. (2021). A novel non-invasive estimation of respiration rate from motion corrupted photoplethysmograph signal using machine learning model. IEEE Access.

[36] Biswas D., Everson L., Liu M., Panwar M., Verhoef B.-E., Patki S., Kim C.H., Acharyya A., Van Hoof C., Konijnenburg M. (2019). CorNET: Deep learning framework for PPG-based heart rate estimation and biometric identification in ambulant environment. IEEE Trans. Biomed. Circuits Syst..

[37] Shin H.C., Roth H.R., Gao M., Lu L., Xu Z., Nogues I., Yao J., Mollura D., Summers R.M. (2016). Deep convolutional neural networks for computer-aided detection: CNN architectures, dataset characteristics and transfer learning. IEEE Trans. Med. Imaging.

[38] Schäck T., Muma M., Zoubir A.M. Computationally efficient heart rate estimation during physical exercise using photoplethysmographic signals. Proceedings of the 2017 25th European Signal Processing Conference (EUSIPCO).

[39] Chowdhury S.S., Hyder R., Hafiz M.S.B., Haque M.A. (2016). Real-time robust heart rate estimation from wrist-type PPG signals using multiple reference adaptive noise cancellation. IEEE J. Biomed. Health Inform..

[40] Yu S.-N., Wang C.-S., Chang Y.P. (2023). Heart Rate Estimation from Remote Photoplethysmography Based on Light-weight U-Net and Attention Modules. IEEE Access.

[41] Kao Y.-H., Chao P.C.-P., Wey C.-L. (2018). Design and validation of a new PPG module to acquire high-quality physiological signals for high-accuracy biomedical sensing. IEEE J. Sel. Top. Quant..

[42] Abreu S. The Pulse in the Wrist Is Felt Over Which Artery?. https://socratic.org/questions/the-pulse-in-the-wrist-is-felt-over-which-artery.

[43] Peller V. (2003). An Introduction to Hankel Operators. Hankel Operators and Their Applications.

[44] Banerjee S., Roy A. (2014). Linear Algebra and Matrix Analysis for Statistics.

[45] Rodgers J.L., Nicewander W.A. (1988). Thirteen ways to look at the correlation coefficient. Am. Stat..

[46] Motin M.A., Karmakar C.K., Palaniswami M. (2019). PPG derived heart rate estimation during intensive physical exercise. IEEE Access.

